# Treatment of Blind Abscesses

**Published:** 1887-04

**Authors:** Louis Ottofy

**Affiliations:** Chicago.


ARTICLE V.

### TREATMENT OF BLIND ABSCESSES.

BY DR. LOUIS OTTOFY, CHICAGO.

The treatment of blind abscesses and the filling of root-canals and cavities with plastic material, all to be done at one sitting, naturally finds much opposition, it being a radical departure from common practice; however, the remedies now at hand for these purposes are such that in many instances it is justifiable.

Unless free access can be gained to all of the roots, the method is not to be relied on, as cleanliness is the cardinal principle of success. The rubber dam should be adjusted, and the *debris* from the cavity entirely removed before any attempt is made to enter the pulp-chamber. The root-canal once opened, they should never be bored, reamed, or any effort made to enlarge them; but instead, a good supply of fine piano wire instruments should be on hand. The canals are first *thoroughly* cleansed with ether and chloroform on cotton, never using such large pieces as to cause a pumping action toward the end of the root. These washings should be continued till neither odor or color is perceptible; however, in roots where the apex is large, which is readily ascertained by the experienced hand, the cotton receives a slight yellowish tinge which does not cease, but this is no bar to proceeding with the treatment. After thorough cleansing, the peroxide of hydrogen is forced in, then the root-canals are again thoroughly dried, and a solution of bichlorate of mercury (1 to 1,000) is forced into and beyond the roots. The root-canals are again thoroughly dried, and with cotton moistened with eucalyptol and dipt into iodoform. These two powerful remedies are forced to occupy every available space in and beyond the canals, and while in that condition, the introduction of a solution of gutta-percha in chloroform, with iodoform (1 ounce of gutta-percha solution, 1 dram of iodoform), is immediately proceeded with. The root one-third or two-thirds filled with it, a cone of oxyphosphate is made, which acting as a piston, is forced into the canals, driving the gutta-percha beyond the root (possibly), at any rate, into every part. The filling (of gold, if not a large cavity), or with any of the plastics, may be immediately introduced. An application of a counter-irritant to the gums is then indicated, which may be either a mixture of equal parts of tincture of iodine and tincture of aconite root; or an iodine paint, which is iodine dissolved in alcohol, four times the strength of the officinal preparation. That either may be effective, the tissue to which this is applied must be dry. The patient is instructed to return within twenty-four hours if troubled. As a general rule, inflammation, sometimes severe, of three or four hours' duration, will follow this treatment. Three precautions should be observed.

1st. Do not select a patient who is lymphatic, anemic, and of such sluggish constitution that the system itself is not properly supported.

2d. Use nothing but pure and reliable remedies.

3d. Each step must be thoroughly performed before another is taken.—*Items of Interest.*

